# Interplay between past market correlation structure changes and future volatility outbursts

**DOI:** 10.1038/srep36320

**Published:** 2016-11-18

**Authors:** Nicoló Musmeci, Tomaso Aste, T. Di Matteo

**Affiliations:** 1 Department of Mathematics, King’s College London, The Strand, London, WC2R 2LS, UK.; 2 Department of Computer Science, UCL, Gower Street, London, WC1E 6BT, UK; 3 Systemic Risk Centre, London School of Economics and Political Sciences, London, WC2A2AE, UK

## Abstract

We report significant relations between past changes in the market correlation structure and future changes in the market volatility. This relation is made evident by using a measure of “correlation structure persistence” on correlation-based information filtering networks that quantifies the rate of change of the market dependence structure. We also measured changes in the correlation structure by means of a “metacorrelation” that measures a lagged correlation between correlation matrices computed over different time windows. Both methods show a deep interplay between past changes in correlation structure and future changes in volatility and we demonstrate they can anticipate market risk variations and this can be used to better forecast portfolio risk. Notably, these methods overcome the curse of dimensionality that limits the applicability of traditional econometric tools to portfolios made of a large number of assets. We report on forecasting performances and statistical significance of both methods for two different equity datasets. We also identify an optimal region of parameters in terms of True Positive and False Positive trade-off, through a ROC curve analysis. We find that this forecasting method is robust and it outperforms logistic regression predictors based on past volatility only. Moreover the temporal analysis indicates that methods based on correlation structural persistence are able to adapt to abrupt changes in the market, such as financial crises, more rapidly than methods based on past volatility.

Forecasting changes in volatility is essential for risk management, asset pricing and scenario analysis. Indeed, models for describing and forecasting the evolution of volatility and covariance among financial assets are widely applied in industry^1^,^2^,^3^,^4^. Among the most popular approaches are worth mentioning the multivariate extensions of GARCH^5^, the stochastic covariance models^6^ and realized covariance^7^. However most of these econometrics tools are not able to cope with more than few assets, due to the curse of dimensionality and the increase in the number of parameters^1^, limiting their insight into the volatility evolution to baskets of few assets only. This is unfortunate, since gathering insights into systemic risk and the unfolding of financial crises require modelling the evolution of entire markets which are composed by large numbers of assets^1^.

We suggest to use network filtering^8^,^9^,^10^,^11^,^12^,^13^,^14^ and metacorrelation as valuable tools to overcome this limitation. Correlation-based filtering networks are tools which have been widely applied to filter and reduce the complexity of covariance matrices made of large numbers of assets (of the order of hundreds), representative of entire markets. This strand of research represents an important part of the Econophysics literature and has given important insights for risk management, portfolio optimization and systemic risk regulation^15^,^16^,^17^,^18^,^19^,^20^.

The volatility of a portfolio depends on the covariance matrix of the corresponding assets^21^. Therefore, the latter can provide insights into the former. In this work we elaborate on this connection showing that correlation matrices can be used to predict variations of volatility. The approach we propose exploits network filtering to explicitly predict future volatility of markets made of hundreds of stocks. This is quite an innovative use of correlation-based networks, which have been so far mostly used for descriptive analyses, with the connections with risk forecasting being mostly overlooked. Some works have shown that is possible to use dimensionality reduction techniques, such as spectral methods^22^, as early-warning signals for systemic risk^23^,^24^: however these approaches, although promising, do not provide proper forecasting tools, as they are affected by high false positive ratios and are not designed to predict a specific quantity.

We quantify the rate of change in the structure of the market correlation matrix by using two tools: (1) the “correlation structure persistence” 〈*ES*〉 which is a measure of similarity between past correlation structures computed from network filtering; (2) the metacorrelation, that is the correlation between correlation matrices at different times; we discuss performances, advantages and disadvantages of the two approaches. We show how such measures exhibit significant predicting power on the market volatility, providing a tool to forecast it. We assess the reliability of these forecasting through out-of-sample tests on two different equity datasets.

The rest of this paper is structured as follows: we first describe the two datasets we have analysed and we introduce the correlation structure persistence and metacorrelation; then we show how our analyses point out a strong interdependence between correlation structure persistence and future changes in the market volatility; moreover, we describe how this result can be exploited to provide a forecasting tool useful for risk management, by presenting out-of-sample tests and false positive analysis; then we investigate how the forecasting performance changes in time; finally we discuss our findings and their theoretical implications.

## Results

We have analysed two different datasets of equity data. The first set (NYSE dataset) is composed by daily closing prices of *N* = 342 US stocks traded in New York Stock Exchange, covering 15 years from 02/01/1997 to 31/12/2012. The second set (LSE dataset) is composed by daily closing prices of *N* = 214 UK stocks traded in the London Stock Exchange, covering 13 years from 05/01/2000 to 21/08/2013. All stocks have been continuously traded throughout these periods of time. These two sets of stocks have been chosen in order to provide a significant sample of the different industrial sectors in the respective markets.

For each asset *i (i* = 1, ..., *N*) we have calculated the corresponding daily log-return *r*_*i*_(*t*) = log (*P*_*i*_(*t*)) − log (*P*_*i*_(*t* − 1)), where *P*_*i*_(*t*) is the asset *i* price at day *t*. The market return *r*_*M*_(*t*) is defined as the average of all stocks returns: *r*_*M*_(*t*) = 1/*N*∑_*i*_*r*_*i*_(*t*). In order to calculate the correlation between different assets we have then analysed the observations by using *n* moving time windows, *T*_*a*_ with *a* = 1, ..., *n*. Each time window contains *θ* observations of log-returns for each asset, totaling to *N* × *n* observations. The shift between adjacent time windows is fixed to *dT* = 5 trading days: therefore, adjacent time windows share a significant number of observations. We have calculated the correlation matrix within each time window, {*ρ*_*ij*_(*T*_*a*_)}, by using an exponential smoothing method^25^ that allows to assign more weight on recent observations. The smoothing factor of this scheme has been chosen equal to *θ*/3 according to previously established criteria^25^.

### A measure of correlation structure persistence with filtering networks

From each correlation matrix {*ρ*_*ij*_(*T*_*a*_)} we computed the corresponding Planar Maximally Filtered Graph (PMFG)^26^ which is one realization of a broader class of filtering networks associating a sparse graph to a correlation matrix^27^. Specifically, the PMFG is a sparse network representation of the correlation matrix that retains only a subset of most significant entries, selected through the topological criterion of being maximally planar^9^. In general, any correlation matrix can be represented as a graph, where each node is an asset and each link between two nodes represents the correlation between them. From this network, which is typically fully connected, several different planar graphs can be extracted as subgraphs: a graph is planar if it can be embedded on a plane without link crossing^26^. The Planar Maximally Filtered Graph (PMFG) is the planar graph associated with the original correlation matrix which maximizes the sum of weights accordingly with a given greedy algorithm^26^. The PMFG can be seen as a generalization of the Minimum Spanning Tree: the PMFG is able to retain a higher amount of information^9^, having a less strict topology constraint allowing to keep a larger number of links. Moreover, the MST is a subgraph of PMFG. In terms of computational complexity the algorithm that builds PMFG is *O*(*N*^3^); recently^27^ a new algorithm has been proposed, able to build a chordal planar graph (called Triangulated Maximally Filtered Graph, TMFG) with an execution time *O*(*N*^2^), making possible a much higher scalability and the application to Big Data^27^. Such networks have been shown to provide a deep insight into the dependence structure of financial assets^9^,^10^,^28^.

**Figure 1:**
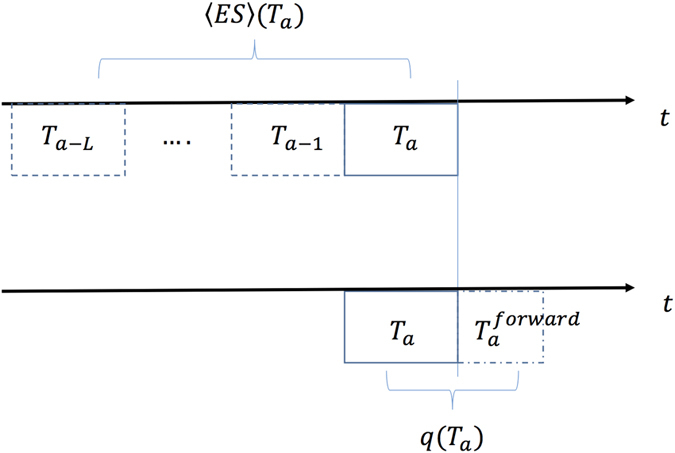
Scheme of time windows setting for 〈*ES*〉(*T*_*a*_) and *q*(*T*_*a*_) calculation. *T*_*a*_ is a window of length *θ*. The correlation structure persistence 〈*ES*〉(*T*_*a*_) (upper axis) is computed by using data in *T*_*a*_ and in the first *L* time windows before *T*_*a*_. The volatility ratio *q*(*T*_*a*_) is computed by using data in *T*_*a*_ and in the future time window 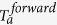. In the upper axis the time windows are actually overlapping, but they are here represented as disjoint for the sake of simplicity.

Once the *n* PMFGs, *G*(*T*_*a*_) with *a* = 1, ..., *n*, have been computed we have calculated a measure that monitors the correlation structure persistence, based on a measure of PMFG similarity. This measure, that we call *ES*(*T*_*a*_, *T*_*b*_), computes the edges in common between a PMFG computed over the time-windows *T*_*a*_ and *T*_*b*_ of length *θ*. Its average over a set of *L* windows *T*_*b*_ (see Fig. 1 and Eqs 2 and 3 in Methods) is denoted with 〈*ES*〉(*T*_*a*_). This measure uses past data only and indicates how slowly the correlation structure measured at time window *T*_*a*_ is differentiating from structures associated to previous time windows.

**Figure 2:**
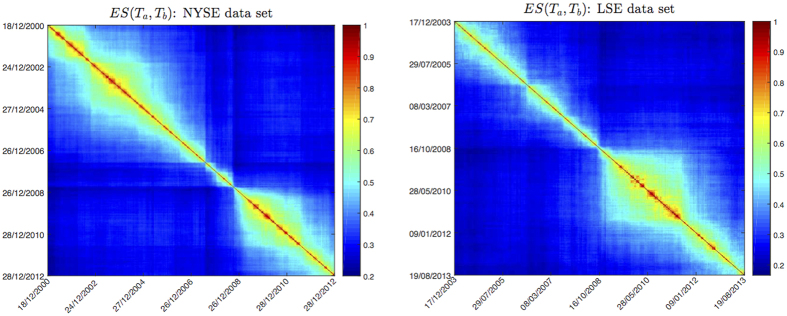
*ES*(*T*_*a*_, *T*_*b*_) matrices for *θ* = 1000, for NYSE (left) and LSE dataset (right). A block-like structure can be observed in both datasets, with periods of high structural persistence and other periods whose correlation structure is changing faster. The 2007–2008 financial crisis marks a transition between two main blocks of high structural persistence.

In Fig. 2 we show the *ES*(*T*_*a*_, *T*_*b*_) matrices (Eq. 3) for the NYSE and LSE dataset, for *θ* = 1000. We can observe a block structure with periods of high structural persistence and other periods whose correlation structure is changing faster. In particular two main blocks of high persistence can be found before and after the 2007–2008 financial crisis; a similar result was found in a previous work^20^ with a different measure of similarity. These results are confirmed for all values of *θ* considered.

### A measure of correlation structure persistence with metacorrelations

**Figure 3:**
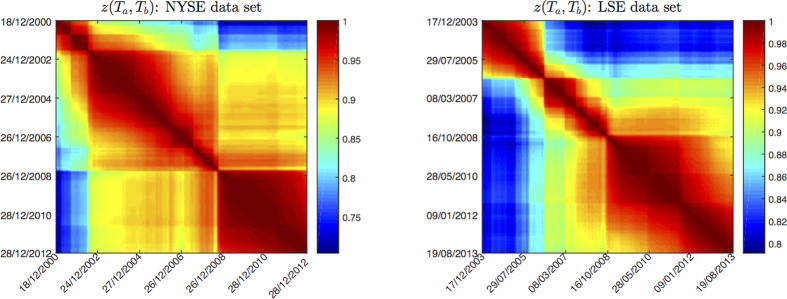
*z*(*T*_*a*_, *T*_*b*_) matrices for *θ* = 1000, for NYSE (left) and LSE dataset (right). A block-like structure can be observed in both datasets, with periods of high structural persistence and other periods whose correlation structure is changing faster. The blocks of high similarity show higher compactness than in Fig. 2.

We have considered a more direct measure of correlation structure persistence, the metacorrelation *z*(*T*_*a*_, *T*_*b*_), that is the Pearson correlation computed between the coefficients of correlation matrices at *T*_*a*_ and *T*_*b*_ (see Methods for more details). Such measure does not make use of any filtering network. Let us note that, although conceptually simpler, this measure requires an equivalent computational complexity (*O*(*N* ^2^)) than 〈*ES*〉(*T*_*a*_) (when computed with the TMFG approach^27^) but it requires a larger memory usage. Figure 3 displays the similarity matrices obtained with this measure for NYSE and LSE datasets: we can observe again block-like structures, that however carry different information from the *ES*(*T*_*a*_, *T*_*b*_) in Fig. 2; in particular, blocks show higher intra-similarity and less structure. Similarly to the construction of *ES*(*T*_*a*_). we have defined 〈*z*〉(*T*_*a*_) as the weighted average over *L* past time windows (see Methods Eqs 2 and 5).

### A forward looking measure of volatility: volatility ratio

The volatility ratio *q*(*T*_*a*_)^29^,^30^ is a foward-looking measure that, at each time window *T*_*a*_, compute the ratio between the estimated volatility in the following time-window 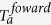 and the one estimated from *T*_*a*_. Unlike 〈*ES*〉(*T*_*a*_) or 〈*z*〉(*T*_*a*_) the value of *q*(*T*_*a*_) is not known at the end of *T*_*a*_ as it requires information from the next time-window. Figure 1 shows a graphical representation of the time window set-up.

### Interplay between correlation structure persistence and volatility ratio

To investigate the relation between 〈*ES*〉(*T*_*a*_) and *q*(*T*_*a*_) we have calculated the two quantities with different values of *θ* and *L* in Eqs 2 and 6, to assess the robustness against these parameters. Specifically, we have used *θ* ∈ (250, 500, 750, 1000) trading days, that correspond to time windows of length 1, 2, 3 and 4 years respectively; *L* ∈ (10, 25, 50, 100), that correspond (given *dT* = 5 trading days) to an average in Eq. 2 reaching back to 50, 125, 250 and 500 trading days respectively. *θ*_*forward*_ has been chosen equal to 250 trading days (one year) for all the analysis.

**Figure 4:**
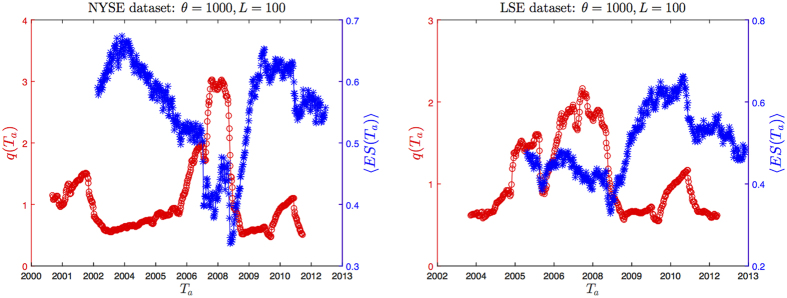
〈*ES*〉(*T*_*a*_) and *q*(*T*_*a*_) signals represented for *θ* = 1000 and *L* = 100, for both NYSE (left graph) and LSE (right graph) datasets. It is evident the anticorrelation between the two signals. The financial crisis triggers a major drop in the structural persistence and a corresponding peak in *q*(*T*_*a*_).

In Fig. 4 we show 〈*ES*〉(*T*_*a*_) and *q*(*T*_*a*_) as a function of time, for *θ* = 1000 and *L* = 100. As expected, main peaks of *q*(*T*_*a*_) occur during the months before the most turbulent periods in the stock market, namely the 2002 market downturn and the 2007–2008 credit crisis. Interestingly, the corresponding 〈*ES*〉(*T*_*a*_) seems to follow a specular trend. This is confirmed by explicit calculation of Pearson correlation between the two signals, reported in Tables 1 and 2: as one can see, for all combinations of parameters the correlation is negative.

In order to check the significance of this anticorrelation we cannot rely on standard tests on Pearson coefficient, such as Fisher transform^31^, as they assume i.i.d. series^32^. Both 〈*ES*〉(*T*_*a*_) and *q*(*T*_*a*_) are instead strongly autocorrelated, due to the significant overlapping between adjacent time windows. In Supplementary Material, Fig. S3, the autocorrelation of *q*(*T*_*a*_) is analysed at different lags. Therefore we have calculated confidence intervals by performing a block bootstrapping test^33^. This is a variation of the bootstrapping test^34^, conceived to take into account the autocorrelation structure of the bootstrapped series. The only free parameter in this method is the block length, that we have chosen applying the optimal selection criterion proposed in literature^35^: such criterion is adaptive on the autocorrelation strength of the series as measured by the correlogram. We have found, depending on the parameters *θ* and *L*, optimal block lengths ranging from 29 to 37, with a mean of 34 (corresponding to 170 trading days). By performing block bootstrapping tests we have therefore estimated confidence intervals for the true correlation between 〈*ES*〉(*T*_*a*_) and *q*(*T*_*a*_); in Tables 1 and 2 correlations whose 95% and 99% confidence intervals (CI) do not include zero are marked with one and two stars respectively. As we can see, 14 out of 16 correlation coefficients are significantly different from zero within 95% CI in the NYSE dataset, and 12 out of 16 in the LSE dataset. For what concerns the 99% CI, we observe 13 out of 16 for the NYSE and 9 out of 16 for the LSE dataset. Non-significant correlations appear only for *θ* = 250, suggesting that this length is too small to provide a reliable measure of structural persistence. Very similar results are obtained by using Minimum Spanning Tree (MST)^36^ instead of PMFG as network filtering.

Given the interpretation of 〈*ES*〉(*T*_*a*_) and *q*(*T*_*a*_) given above, anticorrelation implies that an increase in the “speed” of correlation structure evolution (low 〈*ES*〉(*T*_*a*_)) is likely to correspond to underestimation of future market volatility from historical data (high *q*(*T*_*a*_)), whereas when the structure evolution “slows down” (high 〈*ES*〉(*T*_*a*_)) there is indication that historical data is likely to provide an overestimation of future volatility. This means that we can use 〈*ES*〉(*T*_*a*_) as a valuable predictor of current historical data reliability. This result is to some extent surprising as 〈*ES*〉(*T*_*a*_) is derived from PMFGs topology, which in turns depends only on the ranking of correlations and not on their actual value: yet, this information provides meaningful information about the future market volatility and therefore about the future covariance.

In Tables 3 and 4 we show the correlation between 〈*z*〉(*T*_*a*_) and *q*(*T*_*a*_). As we can see, although an anticorrelation is present for each combination of parameters *θ* and *L*, correlation coefficients are systematically closer to zero than in Tables 1 and 2, where 〈*ES*〉(*T*_*a*_) was used. Moreover the number of significant Pearson coefficients, according to the block bootstrapping, decreases to 12 out of 16 in NYSE and to 10 out of 16 in LSE dataset. Since 〈*z*〉(*T*_*a*_) does not make use of PMFG, this result suggests that the filtering procedure associated to correlation-based networks is enhancing the relation between past correlation structure and future volatility changes. We shall however see in the next session that for forecasting purposes network filtering might not be always beneficial.

### Forecasting volatility with correlation structure persistence

In this section we evaluate how well the correlation structure persistence 〈*ES*〉(*T*_*a*_) can forecast the future through its relation with the forward-looking volatility ratio *q*(*T*_*a*_). In particular we focus on estimating whether *q*(*T*_*a*_) is greater or less than 1: this information, although less complete than a precise estimation of *q*(*T*_*a*_), gives us an important insight into possible overestimation (*q*(*T*_*a*_) < 1) or underestimation (*q*(*T*_*a*_) > 1) of future volatility. The equivalent assessment of forecasting power of 〈*z*〉(*T*_*a*_) is reported in Supplementary Material (Tables S1–S4 and Fig. S1).

We have proceeded as follows. Given a choice of parameters *θ* and *L*, we have calculated the corresponding set of pairs {〈*ES*〉(*T*_*a*_), *q*(*T*_*a*_)}, with *a* = 1, ..., *n*. Then we have defined *Y*(*T*_*a*_) as the categorical variable that is 0 if *q*(*T*_*a*_) < 1 and 1 if *q*(*T*_*a*_) > 1. Finally we have performed a logistic regression of *Y*(*T*_*a*_) against 〈*ES*〉(*T*_*a*_): namely, we assume that^37^:


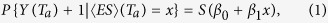


where *S*(*t*) is the sigmoid function 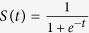
38; we estimate parameters *β*_0_ and *β*_1_ from the observations {〈*ES*〉(*T*_*a*_), *q*(*T*_*a*_)}_*a*=1, ... ,*n*_ through Maximum Likelihood^39^.

**Figure 5:**
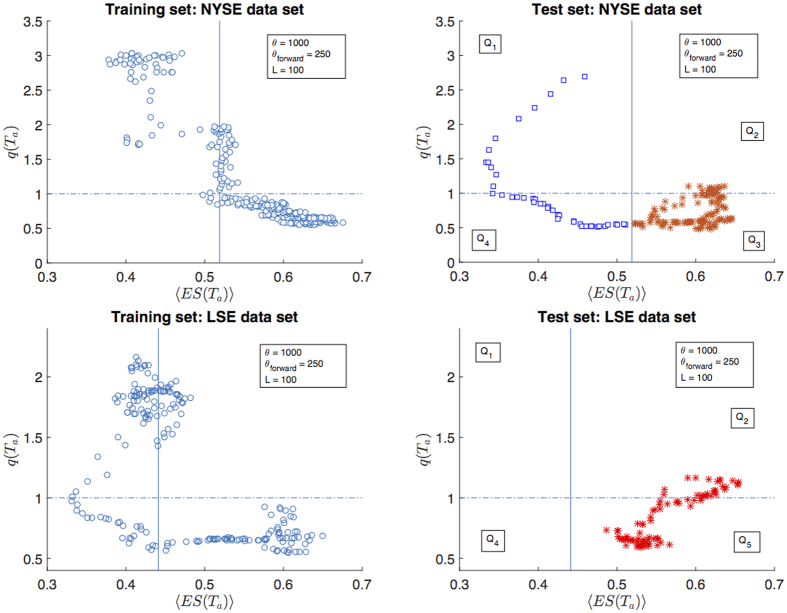
Partition of data into training (left graphs) and test (right graphs) set. Training sets are used to regress *Y*(*T*_*a*_) against 〈*ES*〉(*T*_*a*_), in order to estimate the coefficents in the logistic regression and therefore identify the regression threshold, shown as a vertical continuous line. The test sets are used to test the forecasting performance of such regression on a subset of data that has not been used for regression; the model predicts *Y*(*T*_*a*_) = 1 (*q*(*T*_*a*_) > 1, blue squares in the figure) if 〈*ES*〉(*T*_*a*_) is less than the regression threshold, and *Y*(*T*_*a*_) = 0 (*q*(*T*_*a*_) < 1, red stars in the figure) otherwise.

Once the model has been calibrated, given a new observation 〈*ES*〉(*T*_*n*+1_) = *x* we have predicted *Y*(*T*_*n*+1_) = 1 if *P*{*Y*(*T*_*n*+1_) = 1|〈*ES*〉(*T*_*n*+1_) = *x*} > 0.5, and *Y*(*T*_*n*+1_) = 0 otherwise. This classification criterion, in a case with only one predictor, corresponds to classify *Y*(*T*_*n*+1_) according to whether 〈*ES*〉(*T*_*n*+1_) is greater or less than a threshold *r* which depends on *β*_0_ and *β*_1_, as shown in Fig. 5 (right graphs) for a particular choice of parameters. Therefore the problem of predicting whether market volatility will increase or decrease boils down to a classification problem^39^ with 〈*ES*〉(*T*_*a*_) as predictor and *Y*(*T*_*a*_) as target variable.

We have made use of a logistic regression because it is more suitable than a polynomial model for dealing with classification problems^37^. Other classification algorithms are available; we have chosen the logistic regression due to its simplicity. We have also implemented the KNN algorithm^39^ and we have found that it provides similar outcomes but worse results in terms of the forecasting performance metrics that we discuss in this section.

We have then evaluated the goodness of the logistic regression at estimating *Y*(*T*_*n*+1_) given a new observation 〈*ES*〉(*T*_*n*+1_). To this end, we have computed three standard metrics for assessing the performance of a classification method: the probability of successful forecasting *P*^+^, the True Positive Rate *TPR* and the False Positive Rate *FPR. P*^+^ represents the expected fraction of correct predictions, *TPR* is the method goodness at identifying true positives (in this case, actual increases in volatility) and *FPR* quantifies the method tendency to false positives (predictions of volatility increase when the volatility will actually decrease): see Methods for more details. Overall these metrics provide a complete summary of the model goodness at predicting changes in the market volatility^37^.

In order to avoid overfitting we have estimated the metrics above by means of an out-of-sample procedure^37^,^39^. We have divided our dataset into two periods, a training set and a test set. In the training set we have calibrated the logistic equation in Eq. 1, estimating the parameters *β*_0_ and *β*_1_; in the test set we have used the calibrated model to measure the goodness of the model predictions by computing the measures of performance in Eqs 9, 10, 11. In Fig. 5 this division is shown for a particular choice of *θ* and *L*, for both NYSE and LSE dataset. In this example the percentage of data included in the test set (let us call it *f*_*test*_) is 30%.

Probabilities of successful forecasting *P*^+^ are reported in Tables 5 and 6, for *f*_*test*_ = 30%. As we can see *P*^+^ is higher than 50% for all combinations of parameters in NYSE dataset, and in almost all combinations for LSE dataset. Stars mark those values of *P*^+^ that are significantly higher than the same probability obtained by using the most recent value of *q* instead of 〈*ES*〉(*T*_*a*_) as a predictor for *q*(*T*_*a*_) in the logistic regression (let us call 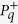 such probability, reported in Tables S5 and 6 in Supplementary Material). Specifically, we have defined a null model where variations from such probability 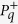 are due to random fluctuations only; given *n* observations, such fluctuations follow a Binomial distribution 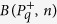, with mean 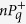 and variance 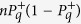. Then p-values have been calculated by using this null distribution for each combination of parameters. This null hypothesis accounts for the predictability of *q*(*T*_*a*_) that is due to the autocorrelation of *q*(*T*_*a*_) only; therefore *P*^+^ significantly higher than the value expected under this hypothesis implies a forecasting power of 〈*ES*〉(*T*_*a*_) that is not explained by the autocorrelation of *q*(*T*_*a*_). From the table we can see that *P*^+^ is significant in 12 out of 16 combinations of parameters for NYSE dataset, and in 13 out of 16 for LSE dataset. This means that correlation persistence is a valuable predictor for future average correlation, able to outperform forecasting method based on past average correlation trends. These results are robust against changes of *f*_*test*_, as long as the training set is large enough to allow an accurate calibration of the logistic regression. We have found this condition is satisfied for *f*_*test*_ < 40%. In Supplementary Material, Fig. S3 reports the autocorrelation of *q*(*T*_*a*_). We also investigated a null model that uses the weighted average version of *q*(*T*_*a*_): results are very similar and reported in Supplementary Material (Tables S9 and 10).

It must be noted that *P*^+^ does not give any information on the method ability to distinguish between true and false positives. To investigate this aspect we need *TPR* and *FPR*. A traditional way of representing both measures from a binary classifier is the so-called “Receiver operating characteristic” (ROC) curve^40^. In a ROC plot, *TPR* is plotted against *FPR* as the discriminant threshold is varied. The discriminant threshold *p*_max_ is the value of the probability in Eq. 1 over which we classify *Y*(*T*_*a*_) = 1: the higher *p*_max_ is, the less likely the method is to classify *Y*(*T*_*a*_) = 1 (in the analysis on *P*^+^ we chose *p*_max_ = 0.5). Ideally, a perfect classifier would yield *TPR* = 1 for all *p*_max_ > 0, whereas a random classifier is expected to lie on the line *TPR* = *FPR*. Therefore a ROC curve which lies above the line *TPR* = *FPR* indicates a classifier that is better than chance at distinguishing true from false positives^37^.

**Figure 6:**
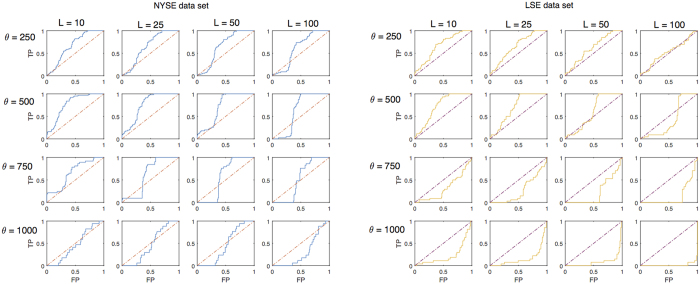
Receiver operating characteristic (ROC) curve. Left graph: True positive rate (TPR) against False positive rate (FPR) as the discriminant threshold *p*_max_ of the classifier is varied, for each combination of parameters *θ* and *L* in the NYSE dataset. The closer the curve is to the upper left corner of each graph, the better is the classifier compared to chance. Right graph: True positive rate (TPR) against False positive rate (FPR) as the discriminant threshold *p*_max_ of the classifier is varied, for each combination of parameters *θ* and *L* in the LSE dataset.

As one can see from Fig. 6, the ROC curve's position depends on the choice of parameters *θ* and *L*. In this respect our classifier performs better for low values of *L* and *θ*. This can be quantified by measuring the area under the ROC curve; such measure, often denoted by AUC^37^, is shown in Tables 7 and 8. For both datasets the optimal choice of parameters is *θ* = 500 and *L* = 10. In Supplementary Material, Tables S7 and 8 and Fig. S4, the ROC curve and AUC values are reported for the case when *q*(*T*_*a*_) is used as predictor, showing that *q*(*T*_*a*_) underperforms 〈*ES*〉(*T*_*a*_) in terms of ROC analysis as well.

We also tested the predictive power of metacorrelations *z*(*T*_*a*_, *T*_*b*_) (see Supplementary Material). By comparing with the results for *ES*(*T*_*a*_, *T*_*b*_) we observed that 〈*z*〉(*T*_*a*_) has almost always a slightly higher *P*^+^ than 〈*ES*〉(*T*_*a*_) in the NYSE dataset, whereas on average it has lower values than 〈*ES*〉(*T*_*a*_) in the LSE dataset (with 9 out of 16 values of *P*^+^ less than the corresponding 〈*ES*〉(*T*_*a*_) probabilities). On the other hand, the ROC analysis shows that the predictor 〈*ES*〉(*T*_*a*_) performs better than 〈*z*〉(*T*_*a*_) in the NYSE dataset and worse in the LSE dataset. Results for forecasting with *z*(*T*_*a*_, *T*_*b*_) are reported in Section S.1 of Supplementary Material (Tables S1 and S4 and Fig. S1). We therefore conclude that the advantage of network filtering in measuring the correlation structure persistence is clear only when it comes to the correlation analysis with *q*(*T*_*a*_), whereas for the forecasting model it might be convenient to use one measure or the other depending on whether one wants to maxmise the probability of forecasting (*P*^+^) or minimise the impact of false positive (ROC analysis).

### Temporal evolution of forecasting performance

In this section we look at how the forecasting performance changes at different time periods. In order to explore this aspect we have counted at each time window *T*_*a*_ the number *N*^+^(*T*_*a*_) of *Y*(*T*_*a*_) predictions (out of the 16 predictions corresponding to as many combinations of *θ* and *L*) that have turned out to be correct; we have then calculated the fraction of successful predictions *n*^+^(*T*_*a*_) as *n*^+^(*T*_*a*_) = *N*^+^(*T*_*a*_)/16. In this way *n*^+^(*T*_*a*_) is a proxy for the goodness of our method at each time window. Logistic regression parameters *β*_0_ and *β*_1_ have been calibrated by using the entire time period as training set, therefore this is an in-sample analysis.

**Figure 7:**
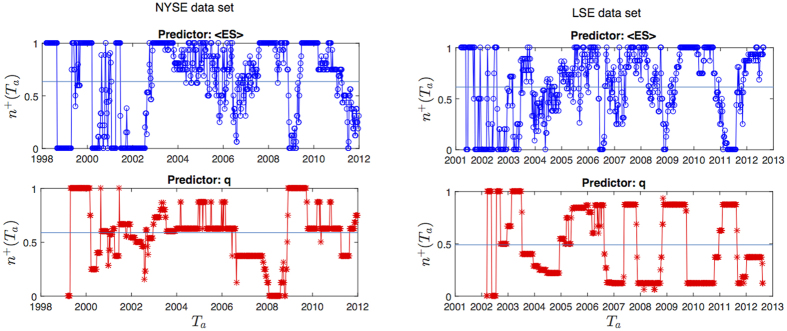
Fraction of successful predictions as a function of time. NYSE (left graph) and LSE dataset (right graph). Forecasting is based on logistic regression with predictor 〈*ES*(*T*_*a*_)〉 (top graphs) and most recent value of *q*(*T*_*a*_) (bottom graphs). Horizontal lines represent the average over the entire period.

In Fig. 7 we show the fraction of successful predictions for both NYSE and LSE datasets (upper graphs, blue circles). For comparison we also show the same measure obtained by using the most recent value of *q*(*T*_*a*_) as predictor (bottom graphs); as in the previous section, it represents a null model that makes prediction by using only the past evolution of *q*(*T*_*a*_). As we can see, both predictions based on 〈*ES*〉(*T*_*a*_) and on past values of *q*(*T*_*a*_) display performances changing in time. In particular *n*^+^(*T*_*a*_) drops just ahead of the main financial crises (the market downturn in March 2002, 2007–2008 financial crisis, Euro zone crisis in 2011); this is probably due to the abrupt increase in volatility that occurred during these events and that the models took time to detect. After these drops though performances based on 〈*ES*〉(*T*_*a*_) recover much more rapidly than those based on past value of *q*(*T*_*a*_). For instance in the first months of 2007 our method shows quite high *n*^+^(*T*_*a*_) (more than 60% of successful predictions), being able to predict the sharp increase in volatility to come in 2008 while predictions based on *q*(*T*_*a*_) fail systematically until 2009. Overall, predictions based on correlation structure persistence appear to be more reliable (as shown by the average *n*^+^(*T*_*a*_) over all time windows, the horizontal lines in the plot) and faster at detecting changes in market volatility. Similar performances to 〈*ES*〉(*T*_*a*_) can be observed when *n*^+^(*T*_*a*_) is calculated using 〈*z*〉(*T*_*a*_) as measure of correlation structure persistence (see Supplementary Material, Fig. S2).

## Discussion

In this paper we have proposed a new tool for forecasting market volatility based on correlation-based information filtering networks, metacorrelation and logistic regression, useful for risk and portfolio management. The advantage of our approach over traditional econometrics tools, such as multivariate GARCH and stochastic covariance models, is the “top-down” methodology that treats correlation matrices as the fundamental objects, allowing to deal with many assets simultaneously; in this way the curse of dimensionality, that prevents e.g. multivariate GARCH to deal with more than few assets, is overcome. We have proven the forecasting power of this tool by means of out-of-sample analyses on two different stock markets; the forecasting performance has been proven to be statistically significant against a null model, outperforming predictions based on past market correlation trends. Moreover we have measured the ROC curve and identified an optimal region of the parameters in terms of True Positive and False Positive trade-off. The temporal analysis indicates that our approach, based on correlation structure persitance, is able to adapt to abrupt changes in the market, such as financial crises, more rapidly than methods based on past volatility.

This forecasting tool relies on an empirical fact that we have reported in this paper for the first time. Specifically, we have shown that there is a deep interplay between future changes in market volatility and the rate of change of the past correlation structure. The statistical significance of this relation has been assessed by means of a block-bootstrapping technique. An analysis based on metacorrelation has revealed that this interplay is better highlighted when filtering based on correlation filtering graphs (Planar Maximally Filtered Graphs) is used to estimate the correlation structure persistence. However, when it comes to forecasting performances, metacorrelation might be preferable to network filtering On the other hand, the use of planar graphs allows a better visualisation of the system, making possible a clearer interpretation of correlation structure changes. We must note that correlation filtering networks retain a much smaller amount of information than the whole correlation matrix and nonetheless reveal larger relations with future volatility changes and comparable forecasting performances. This could be crucial for a possible use of this tools in the context of big-data analytic where several thousands of indicators are simultaneously considered.

This finding sheds new light into the dynamic of correlation. Both metacorrelation and the topology of correlation filtering networks depend on the ranking of the *N*(*N* − 1)/2 pairs of cross-correlations; therefore a decrease in the correlation structure persistence points out a faster change of this ranking. Our result indicates that such increase is typically followed by a rise in the market volatility, whereas decreases are followed by drops. A possible interpretation of this is related to the dynamics of risk factors in the market. Indeed higher volatility in the market is associated to the emergence of a (possibly new) risk factor that makes the whole system unstable; such transition could be anticipated by a quicker change of the correlation ranking, triggered by the still emerging factor and revealed by the correlation structure persistence. Such persistence can therefore be a powerful tool for monitoring the emergence of new risks, valuable for a wide range of applications, from portfolio management to systemic risk regulation. Moreover this interpretation would open interesting connections with those approaches to systemic risk that make use of Principal Component Analysis, monitoring the emergence of new risk factors by means of spectral methods^23^,^24^. We plan to investigate all these aspects in a future work.

## Methods

### Correlation structure persistence with filtering networks 〈*ES*〉(*T*_*a*_)

We define the correlation structure persistence at time *T*_*a*_ as:


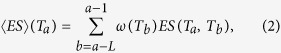


where 
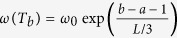
 is an exponential smoothing factor, *L* is a parameter and *ES*(*T*_*a*_, *T*_*b*_) is the fraction of edges in common between the two PMFGs *G*(*T*_*a*_) and *G*(*T*_*b*_), called “edge survival ratio”^15^. In formula, *ES*(*T*_*a*_,*T*_*b*_) reads:


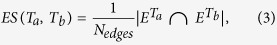


where *N*_*edges*_ is the number of edges (links) in the two PMFGs (constant and equal to 3*N* − 6 for a PMFG^26^), and 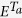 (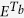) represents the edge-sets of PMFG at *T*_*a*_ (*T*_*b*_). The correlation structure persistence 〈*ES*〉(*T*_*a*_) is therefore a weighted average of the similarity (as measured by the edge survival ratio) between *G*(*T*_*a*_) and the first *L* previous PMFGs, with an exponential smoothing scheme that gives more weight to those PMFGs that are closer to *T*_*a*_. The parameter *ω*_0_ in Eq. 2 can be calculated by imposing 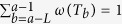. Intuitively, 〈*ES*〉(*T*_*a*_) measures how slowly the change of correlation structure is occurring in the near past of *T*_*a*_.

### Correlation structure persistence with metacorrelation 〈*z*〉(*T*_*a*_)

Given two correlation matrices {*ρ*_*ij*_(*T*_*a*_)} and {*ρ*_*ij*_(*T*_*b*_)} at two different time windows *T*_*a*_ and *T*_*b*_, their metacorrelation *z*(*T*_*a*_,*T*_*b*_) is defined as follows:


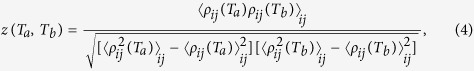


where 〈...〉_*ij*_ is the average over all couples of stocks *i, j*. Similarly to Eq. 2 we have then defined *z*(*T*_*a*_) as the weighted average over *L* past time windows:


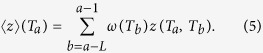


### Volatility ratio *q*(*T*_*a*_)

In order to quantify the agreement between the estimated and the realized risk we here make use of the volatility ratio, a measure which has been previously used^29^,^41^ for this purpose and defined as follows:


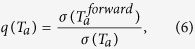


where 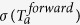 is the realized volatility of the average market return *r*_*M*_(*t*) computed on the time window 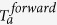; *σ*(*T*_*a*_) is the estimated volatility of *r*_*M*_(*t*) computed on time window *T*_*a*_, by using the same exponential smoothing scheme^25^ described for the correlation {*ρ*_*ij*_(*T*_*a*_)}. Specifically, 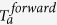 is the time window of length *θ*_*forward*_ that follows immediately *T*_*a*_: if *t*_*θ*_ is the last observation in *T*_*a*_, 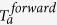 covers observations from *t*_*θ*+1_ to 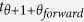 (Fig. 1). Therefore the ratio in Eq. 6 estimates the agreement between the market volatility estimated with observations in *T*_*a*_ and the actual market volatility observed over an investment in the *N* assets over 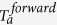. If *q*(*T*_*a*_) > 1, then the historical data gathered at *T*_*a*_ has underestimated the (future) realized volatilty, whereas *q*(*T*_*a*_) < 1 indicates overestimation. Let us stress that *q*(*T*_*a*_) provides an information on the reliability of the covariance estimation too, given the relation between market return volatility and covariance^21^:


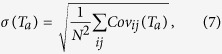



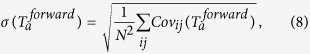


where *Cov*_*ij*_(*T*_*a*_) and 
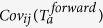
 are respectively the estimated and realized covariances.

### Measures of classification performance

With reference to Fig. 5b,d, let us define the number of observations in each quadrant *Q*_*i*_ (*i* = 1, 2, 3, 4) as |*Q*_*i*_|. In the terminology of classification techniques^39^, |*Q*_1_| is the number of True Positive (observations for which the model correctly predicted *Y*(*T*_*a*_) = 1), |*Q*_3_| is the number of True Negative (observations for which the model correctly predicted *Y*(*T*_*a*_) = 0), |*Q*_2_| the number of False Negative (observations for which the model incorrectly predicted *Y*(*T*_*a*_) = 0) and |*Q*_4_| the number of False Positive (observations for which the model incorrectly predicted *Y*(*T*_*a*_) = 1). We have then computed the following measures of quality of classification, that are the standard metrics for assessing the performances of a classification method^39^:

- **Probability of successful forecasting (P^+^)^39^:** represents the method probability of a correct prediction, expressed as fraction of observed 〈*ES*〉(*T*_*a*_) values through which the method has successfully identified the correspondent value of *Y*(*T*_*a*_). In classification problems, sometimes, the error rate *I* is used^37^, which is simply *I* = 1 − *P*^+^. *P*^+^ is computed as follows: 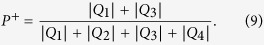
- **True Positive Rate (TPR)^39^:** it is the probability of predicting *Y*(*T*_*a*_) = 1, conditional to the fact that the real *Y*(*T*_*a*_) is indeed 1 (that is, to predict an increase in volatility when the volatility will indeed increase); it represents the method sensitivity to increase in volatility. It is also called “recall”^37^. In formula:

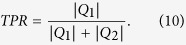
- **False Positive Rate (FPR)^39^:** it is the probability of predicting *Y*(*T*_*a*_) = 1, conditional to the fact that the real *Y*(*T*_*a*_) is instead 0 (that is, to predict an increase in volatility when the volatility will actually decrease). It is also called “1-specificity”^37^. In formula: 
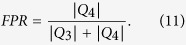

## Additional Information

**How to cite this article**: Musmeci, N. *et al*. Interplay between past market correlation structure changes and future volatility outbursts. *Sci. Rep.* **6**, 36320; doi: 10.1038/srep36320 (2016).

**Publisher’s note**: Springer Nature remains neutral with regard to jurisdictional claims in published maps and institutional affiliations.

## Supplementary Material

Supplementary Information

## Figures and Tables

**Table 1:** NYSE dataset: correlation between 〈*ES*〉(*T*_*a*_) and *q*(*T*_*a*_), for different combinations of parameters *θ* and *L*.

		L			
		10	25	50	100
*θ*	250	−0.2129	−0.2224	−0.2997^*^	−0.3498^**^
	500	−0.4276^**^	−0.4683^**^	−0.4945^**^	−0.5354^**^
	750	−0.4994^**^	−0.5499^**^	−0.5837^**^	−0.6018^**^
	1000	−0.5789^**^	−0.6152^**^	−0.6480^**^	−0.6874^**^

Stars mark those correlation coefficients whose confidence interval excludes zero with a 95% (one star) or a 99% confidence (two stars). The confidence intervals are computed from the block-bootstrapped sample.

**p < 0.001, *p < 0.01.

**Table 2:** LSE dataset: correlation between 〈*ES*〉(*T*_*a*_) and *q*(*T*_*a*_), for different combinations of parameters *θ* and *L*.

		L			
		10	25	50	100
*θ*	250	−0.2084^*^	−0.1887^*^	−0.1872	−0.2269^*^
	500	−0.3083^**^	−0.3343^**^	−0.3782^**^	−0.4202^**^
	750	−0.4050^**^	−0.4409^**^	−0.4334^**^	−0.4374^**^
	1000	−0.4552^**^	−0.5285^**^	−0.5480^**^	−0.5227^**^

Stars mark those correlation coefficients whose confidence interval excludes zero with a 95% (one star) or a 99% confidence (two stars). The confidence intervals are computed from the block-bootstrapped sample.

**p < 0.001, *p < 0.01.

**Table 3:** NYSE dataset: correlation between 〈*z*〉(*T*_*a*_) and *q*(*T*_*a*_), for different combinations of parameters *θ* and *L*.

		L			
		10	25	50	100
*θ*	250	−0.0992	−0.0754	−0.1055	−0.1157
	500	−0.2146	−0.2232	−0.2309	−0.2753
	750	−0.2997	−0.3706^*^	−0.4030^*^	−0.4109^*^
	1000	−0.3933^**^	−0.4290^**^	−0.4678^**^	−0.4574^*^

Stars mark those correlation coefficients whose confidence interval excludes zero with a 95% (one star) or a 99% confidence (two stars). The confidence intervals are computed from the block-bootstrapped sample.

**p < 0.001, *p < 0.01.

**Table 4:** LSE dataset: correlation between 〈*z*〉(*T*_*a*_) and *q*(*T*_*a*_), for different combinations of parameters *θ* and *L*.

		L			
		10	25	50	100
*θ*	250	−0.1470	−0.1095	−0.1326	−0.1720
	500	−0.2365^*^	−0.2113	−0.2936^*^	−0.3932^**^
	750	−0.3123^**^	−0.3379^*^	−0.3538^*^	−0.3851^*^
	1000	−0.2917^*^	−0.2954	−0.3163	−0.4192^**^

Stars mark those correlation coefficients whose confidence interval excludes zero with a 95% (one star) or a 99% confidence (two stars). The confidence intervals are computed from the block-bootstrapped sample.

**p < 0.001, *p < 0.01.

**Table 5:** NYSE dataset: Probability of successful forecasting *P* ^+^, for different combinations of parameters *θ* and *L*.

		L			
		10	25	50	100
*θ*	250	0.546	0.560*	0.599**	0.539**
	500	0.704**	0.695**	0.658**	0.605**
	750	0.634*	0.585	0.539	0.708*
	1000	0.704*	0.7638**	0.839**	0.860

Out-of-sample analysis.

**p < 0.001, *p < 0.01.

**Table 6:** LSE dataset: Probability of successful forecasting *P* ^+^, for different combinations of parameters *θ* and *L*.

		L			
		10	25	50	100
*θ*	250	0.616**	0.645**	0.612**	0.568**
	500	0.652**	0.635**	0.598**	0.393
	750	0.651**	0.560**	0.453**	0.412
	1000	0.544**	0.573**	0.706**	0.689

Out-of-sample analysis.

**p < 0.001, *p < 0.01.

**Table 7:** NYSE dataset: Area under the curve (AUC), measured from the ROC curve in Fig. 6.

		L			
		10	25	50	100
*θ*	250	0.669	0.652	0.655	0.616
	500	0.775	0.753	0.710	0.625
	750	0.663	0.6220	0.574	0.520
	1000	0.467	0.470	0.462	0.314

Values greater than 0.5 indicate that the classifier performs better than chance.

**Table 8:** LSE dataset: Area under the curve (AUC), measured from the ROC curve in Fig. 6.

		L			
		10	25	50	100
*θ*	250	0.673	0.658	0.618	0.524
	500	0.727	0.700	0.602	0.431
	750	0.324	0.274	0.234	0.148
	1000	0.233	0.168	0.0918	0.0160

Values greater than 0.5 indicate that the classifier performs better than chance.
