# Supplementary material for: Interplay between past market correlation structure changes and future volatility outbursts
Source: Sci Rep. 2016 Nov 18;6:36320. doi: 10.1038/srep36320 (PMC5114656; doi:10.1038/srep36320)
Supplement: Supplementary Information [file srep36320-s1.pdf]

Supplementary Material for : Interplay between market structure changes and future volatility outbursts

Nicoló Musmeci[1], Tomaso Aste[2,3,*], and T. Di Matteo[1,2]

[1]*Department of Mathematics, King's College London, The Strand, London, WC2R 2LS*
[2]*Department of Computer Science, UCL, Gower Street, London, WC1E 6BT, UK*
[3]*Systemic Risk Centre, London School of Economics and Political Sciences, London, WC2A2AE, UK*
[*]*t.aste@ucl.ac.uk*

S1 Forecasting performance of $\langle z\rangle(T_a)$

In this section we present further analyses on $z(T_a, T_b)$, namely P^+ and ROC. From Tabs. S1-S2 we can see that $\langle z\rangle(T_a)$ has almost always an higher P^+ than $\langle ES\rangle(T_a)$ in the NYSE dataset, whereas on average it has lower values than $\langle ES\rangle(T_a)$ in the LSE dataset (with 7 out of 16 values of P^+ greater than the corresponding $\langle ES\rangle(T_a)$ probabilities). On the other hand, the ROC analysis in Fig. S1 and Tabs. S3 - S4 shows that the predictor $\langle ES\rangle(T_a)$ performs better than $\langle z\rangle(T_a)$ in the NYSE dataset and worse in the LSE dataset. Fig. S2 shows $\langle z\rangle(T_a)$ performances in time.

Table S1: **NYSE dataset: Probability of successful forecasting P^+ using $\langle z\rangle(T_a)$ as a predictor $\langle z\rangle(T_a)$ or $\langle z(T_a, T_b)\rangle$**, for different combinations of parameters θ and L. Out-of-sample analysis.

		L			
		**10**	**25**	**50**	**100**
θ	**250**	0.592*	0.587**	0.632**	0.652**
	**500**	0.732**	0.707**	0.670**	0.671**
	**750**	0.786**	0.736**	0.697**	0.759**
	**1000**	0.886**	0.882**	0.861**	0.860

$^{**}p < 0.001$, $^{*}p < 0.01$,

Table S2: **LSE dataset: Probability of successful forecasting P^+ using $\langle z\rangle(T_a)$ as a predictor**, for different combinations of parameters θ and L. Out-of-sample analysis.

		L			
		**10**	**25**	**50**	**100**
θ	**250**	0.723**	0.342	0.598*	0.522
	**500**	0.701**	0.650**	0.621*	0.589
	**750**	0.674**	0.464	0.256	0.666**
	**1000**	0.236	0.245	0.441	0.689**

$^{**}p < 0.001$, $^{*}p < 0.01$,

Table S3: **NYSE dataset: Area under the curve (AUC) using $\langle z\rangle(T_a)$ as a predictor**, measured from the ROC curve in Fig. S1. Values greater than 0.5 indicate that the classifier performs better than chance.

		L			
		**10**	**25**	**50**	**100**
θ	**250**	0.803	0.700	0.625	0.496
	**500**	0.834	0.761	0.639	0.406
	**750**	0.633	0.549	0.436	0.135
	**1000**	0.413	0.393	0.352	0.107

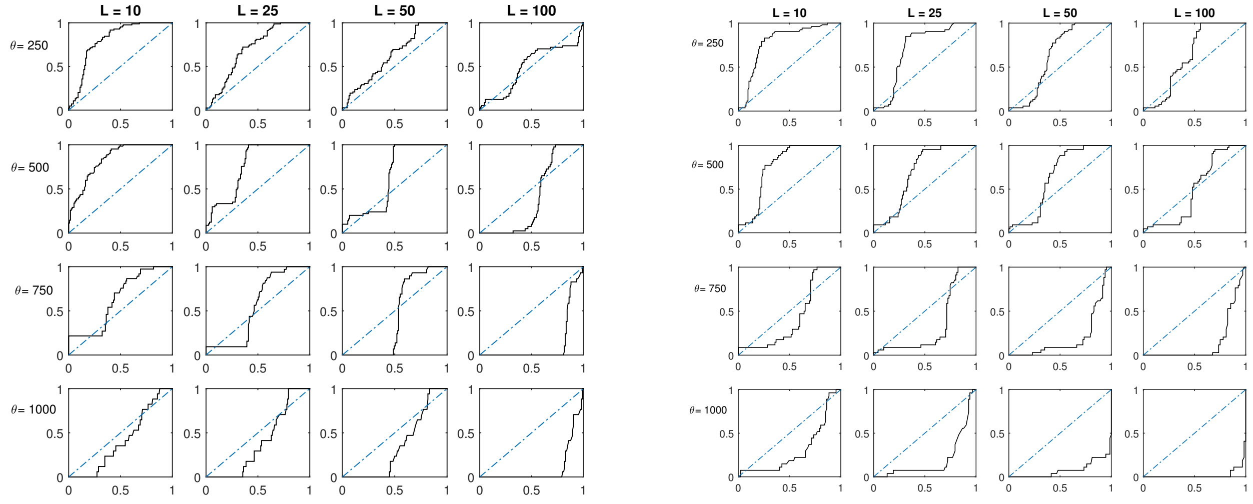

Figure S1: **Receiver operating characteristic (ROC) curve for the NYSE and LSE dataset, by using $\langle z\rangle(T_a)$ as a predictor**. True positive rate (TPR) against False positive rate (FPR) as the discriminant threshold p_{max} of the classifier is varied, for each combination of parameters θ and L in the NYSE dataset. The closer the curve is to the upper left corner of each graph, the better is the classifier compared to chance.

Table S4: **LSE dataset: Area under the curve (AUC) using $\langle z\rangle(T_a)$ as a predictor**, measured from the ROC curve in Fig. S1. Values greater than 0.5 indicate that the classifier performs better than chance.

		L			
		**10**	**25**	**50**	**100**
θ	**250**	0.790	0.714	0.637	0.636
	**500**	0.765	0.694	0.638	0.496
	**750**	0.434	0.338	0.200	0.150
	**1000**	0.309	0.200	0.079	0.022

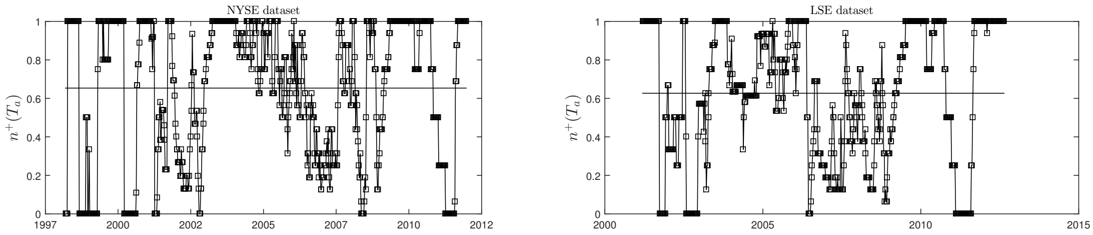

Figure S2: **Fraction of successful predictions as a function of time, using $\langle z\rangle(T_a)$ as a predictor**. NYSE (left graph) and LSE dataset (right graph). Horizontal lines represent the average over the entire period.

S2 Forecasting performance of $q(T_a)$

In this section we report some further analyses on the performances of $q(T_a)$ as a predictor in the logistic regression.

In Fig. S3 we show the autocorrelation of $q(T_a)$ for different lags and different values of θ. As we can see, $q(T_a)$ displays a strong autocorrelation.

In Tabs S5-S6 we report the probabilities P^+ obtained by using $q(T_a)$ as a predictor in the logistic regression. These values are the null probabilities which have been used in Tabs. 5-6 of the paper to compute the p-values.

In Fig. S4 the ROC curves for $q(T_a)$ as a predictor are shown, whereas in Tabs S7 - S8 we report the corresponding AUC values. As we can see $q(T_a)$ performs quite poorly in terms of ROC analysis as well, underperforming the random predictor for almost any choice of the parameters. Specifically, in terms of AUC the $\langle ES\rangle(T_a)$ predictor outperforms $q(T_a)$ for all the combinations of parameters in the NYSE dataset , whereas in the LSE dataset this is true in 8 out of 16 combinations of parameters: the rest are those parameters combination for which both $\langle ES\rangle(T_a)$ and $q(T_a)$ underperform the random predictor.

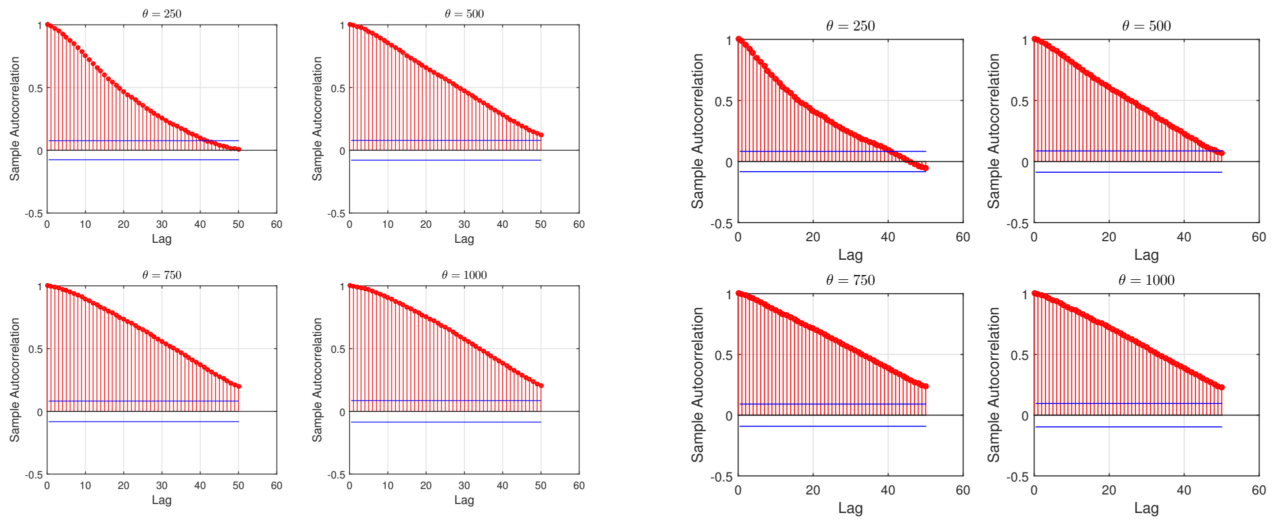

Figure S3: Autocorrelation of $q(T_a)$ at different lags, for each value of θ. Left panel: NYSE dataset. Right panel: LSE dataset.

Table S5: **NYSE dataset: Probability of successful forecasting P^+ using $q(T_a)$ as a predictor**, for different combinations of parameters θ and L. Out-of-sample analysis.

		L			
		**10**	**25**	**50**	**100**
θ	**250**	0.487	0.468	0.420	0.373
	**500**	0.455	0.433	0.403	0.417
	**750**	0.509	0.493	0.503	0.602
	**1000**	0.594	0.643	0.691	0.785

$^{**}p < 0.001$, $^{*}p < 0.01$,

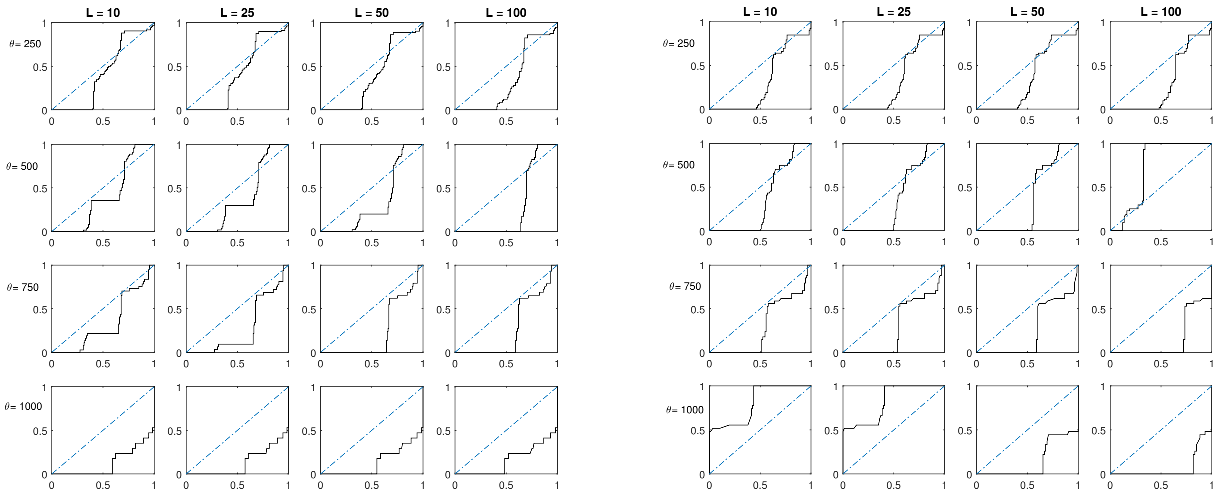

Figure S4: **Receiver operating characteristic (ROC) curve for the NYSE and LSE dataset, by using $q(T_a)$ as a predictor**. True positive rate (TPR) against False positive rate (FPR) as the discriminant threshold p_{max} of the classifier is varied, for each combination of parameters θ and L in the NYSE dataset. The closer the curve is to the upper left corner of each graph, the better is the classifier compared to chance.

Table S6: **LSE dataset: Probability of successful forecasting P^+ using $q(T_a)$ as a predictor**, for different combinations of parameters θ and L. Out-of-sample analysis.

		L			
		**10**	**25**	**50**	**100**
θ	**250**	0.373	0.357	0.438	0.404
	**500**	0.412	0.388	0.335	0.379
	**750**	0.406	0.282	0.293	0.326
	**1000**	0.239	0.247	0.237	0.686

$^{**}p < 0.001$, $^{*}p < 0.01$,

Table S7: **NYSE dataset: Area under the curve (AUC) using $q(T_a)$ as a predictor**, measured from the ROC curve in Fig. S4. Values greater than 0.5 indicate that the classifier performs better than chance.

		L			
		**10**	**25**	**50**	**100**
θ	**250**	0.421	0.410	0.393	0.344
	**500**	0.406	0.386	0.353	0.294
	**750**	0.336	0.283	0.248	0.282
	**1000**	0.128	0.133	0.141	0.160

Table S8: **LSE dataset: Area under the curve (AUC) using $q(T_a)$ as a predictor**, measured from the ROC curve in Fig. S4. Values greater than 0.5 indicate that the classifier performs better than chance.

		L			
		**10**	**25**	**50**	**100**
θ	**250**	0.323	0.335	0.363	0.305
	**500**	0.362	0.376	0.378	0.719
	**750**	0.300	0.302	0.257	0.157
	**1000**	0.806	0.821	0.150	0.074

Finally, in Tabs S9-S10 we report the P^+ probabilities obtained by using a weighted average version of $q(T_a)$, $\langle q \rangle(T_a)$, as predictor. As we can see the values are very close to those in Tabs S5-S6 obtained by using simply $q(T_a)$ as predictor; overall the performance of $q(T_a)$ is not improved. We interpret this as a consequence of the strong autocorrelation of $q(T_a)$, which makes the average $\langle q \rangle(T_a)$ very close to $q(T_a)$, and therefore the two predictors very similar.

Table S9: **NYSE dataset: Probability of successful forecasting P^+ using $\langle q \rangle(T_a)$ as a predictor**, for different combinations of parameters θ and L. Out-of-sample analysis.

		L			
		**10**	**25**	**50**	**100**
θ	**250**	0.481 0.441	0.419	0.373	
	**500**	0.449	0.427	0.415	0.364
	**750**	0.509	0.493	0.509	0.426
	**1000**	0.601	0.643	0.654	0.231

$^{**}p < 0.001$, $^{*}p < 0.01$,

Table S10: **LSE dataset: Probability of successful forecasting P^+ using$\langle q(T_a) \rangle$ as a predictor**, for different combinations of parameters θ and L. Out-of-sample analysis. *7-8 for ES*

		L			
		**10**	**25**	**50**	**100**
θ	**250**	0.367	0.389	0.363	0.564
	**500**	0.328	0.316	0.335	0.448
	**750**	0.242	0.274	0.293	0.663
	**1000**	0.239	0.247	0.267	0.686

$^{**}p < 0.001$, $^{*}p < 0.01$,

S3 Relation between $\langle ES \rangle(T_a)$ and $\sigma(T_a)$

In Fig. S5 we report the scatter plot of $\sigma(T_a)$ against $\langle ES \rangle(T_a)$. As we can see, no clear pattern appears which could explain the relationship between $\langle ES \rangle(T_a)$ and $q(T_a)$ reported in the paper.

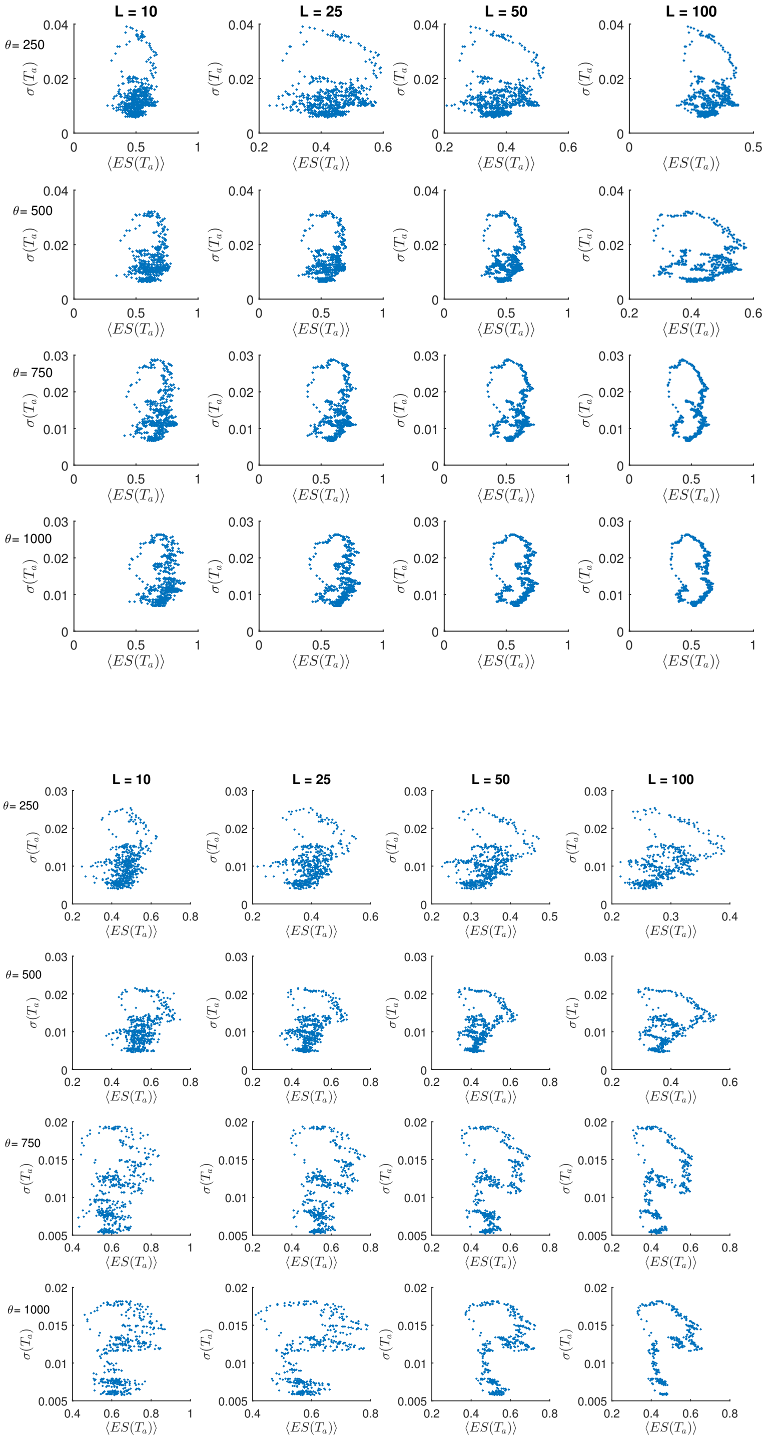

Figure S5: Scatter plot of $\sigma(T_a)$ against $\langle ES \rangle(T_a)$, for each combination of parameters L and θ, for the NYSE (left) and LSE (right) datasets.
